# Research on the healthy life expectancy of older adult individuals in China based on intrinsic capacity health standards and social stratification analysis

**DOI:** 10.3389/fpubh.2023.1303467

**Published:** 2024-01-15

**Authors:** Mengya Liu, Meng Zhang, Jinglei Zhou, Nannan Song, Li Zhang

**Affiliations:** Bengbu Medical University, Bengbu, Anhui, China

**Keywords:** healthy life expectancy, intrinsic capacity, older age, social stratification, health aging

## Abstract

**Background:**

Based on the health standard of intrinsic capacity, this paper conducts an empirical study on the healthy life expectancy of older adult individuals aged 60 and older in China and analyzes the health inequities associated with different social characteristics to provide a reference for improving care for the older adult in China.

**Methods:**

Data from the China Health and Retirement Longitudinal Study from 2011 to 2015 were used to evaluate the intrinsic capacity level of older adult individuals, and the multistate life table method was used to measure the healthy life expectancy of older adult individuals in China with the help of IMaCH software. Based on the theory of social stratification, the health inequality between older adult individuals in different social classes was analyzed in three dimensions: residence, income and education level.

**Results:**

The calculation results show that the average life expectancy of the older adult in China at age 60 is 21.07 years, the healthy life expectancy is 16.89 years, and the healthy life expectancy accounts for 80.2% of the average life expectancy. The healthy life expectancy of older adult individuals with different social characteristics in China shows significant differences, and the healthy life expectancy of older adult individuals who are male, live in urban environments, have high levels of education and have middle- to high-income levels is significantly better than that of older adult individuals who are female, live in rural areas, have low levels of education and income.

**Conclusion:**

Healthy life expectancy measured by intrinsic capacity as the health standard has a certain reference value, which reflects the overall health level of older adult individuals in China and expands the transformation and multidimensional understanding of the healthy thinking of older adult individuals in China. The analysis by social stratification reflects the large health inequities that exist in the older adult population in China.

## Introduction

The aging of the world's population is a serious situation. According to the World Population Prospects report released by the United Nations in 2022, the proportion of the global population aged 60 and older will increase from 13.5% in 2020 to 21.4% by 2050 (1). China is also facing the challenge of a rapidly aging population. In 2020, China's seventh population census reported ~260 million older adult aged 60 and older, accounting for 18.7% of the total population, indicating that China has an aging society (2). The greatest challenge posed by an aging population is the serious and complex health problems of older adult individuals. By the end of 2020, Chinese life expectancy was 77.3 years, while healthy life expectancy was only 69 years, indicating that older adult individuals in China have 8.3 years in which they will experience health problems such as disease, impairment and disability (3). Lifespan only reflects the length of life, and health truly reflects the quality of life. With the development of China's economy and the improvement of living conditions, we are paying more attention to improving the quality of life of older adult individuals, and the primary pursuit is not to “live longer” but to “live healthier and happier.” How to help older adult individuals live longer and healthier lives is the top priority of the Chinese government's work on aging in the new era. The Central Committee of the Communist Party of China and the State Council have issued the “Healthy China 2030” planning outline, which proposes to further promote the strategic theme of “co-construction and sharing, and health for all” (4) with people's health as the center and “healthy life expectancy” as an important monitoring indicator to comprehensively reflect the level of health. Combining the length of life with the quality of life, healthy life expectancy is a comprehensive evaluation index to evaluate the health level of the world population.

One of the focuses of healthy life expectancy research is the selection of health assessment criteria. At present, scholars from all over the world have developed and formulated a variety of health status evaluation indicators based on disease surveillance and health survey data, such as disease burden, disability, ability to perform activities of daily living (ADLs), and health self-assessment, and these indicators are applicable to people of all ages to assess their health. Population aging is a common trend in world population development, the health of the older adult is attracting increasing international attention, and traditional universal health indicators lack characteristics and pertinence for evaluating the health of older adult individuals. Therefore, we need an evaluation index dedicated to evaluating the health of older adult. In 2015, the WHO released the Global Report on Aging and Health, which breaks the traditional concept of focusing on diseases and defines “healthy aging” as the process of developing and maintaining the functional exertion required for healthy life among the older adult from the perspective of functional maintenance (5). Intrinsic capacity (IC) is the basis for functional performance, which refers to the combination of all physical functions and mental power that an individual can use at any time. As a framework for monitoring the health of older adult individuals, intrinsic capacity reflects the overall state of older adult individuals, paying more attention to the various functions that older adult can do as they wish, which they deem important. To translate theoretical models into practice, the constituent elements of intrinsic capacity need to be defined to make assessment, measurement and monitoring operational. After multiregional and multidisciplinary expert consultation, literature review and empirical pilot research, WHO proposed five dimensions of intrinsic capacity, including cognition, motor, vitality, sensory and psychology, which are considered to fully reflect the physical function and brain changes in the framework of healthy aging, representing measurable aging indicators, and can scientifically, comprehensively and standardize the health level of the older adult from different perspectives. To fully reflect the change in global cognition of the health of the older adult, this study will use intrinsic capacity as a health standard to measure the healthy life expectancy of the older adult in China, explore a new measurement index for measuring the healthy life expectancy of older adult, and scientifically, objectively and operably evaluate the health level of the older adult in China.

In the context of the deepening of aging, how to promote “healthy aging” and improve the health level of older adult individuals has become an important issue for government and society (6). In 2019, the Chinese government released the “Opinions of the General Office of the State Council on Promoting the Development of Elderly Care Services,” which pointed out that the current construction of the older adult care service system has achieved remarkable results, but there are still many problems, such as uneven development (6). The health level of the older adult is the basic factor for forming the demand for elder care services and building a social elder care service system, and the health inequality caused by social stratification is an important perspective to consider when exploring the imbalance of elder care service development (7). Social stratification refers to the stratification or difference phenomenon caused by the different social resources of members of society and social groups; Weber identified three basic dimensions of social stratification, namely, wealth, power and prestige (8). Previous studies have shown significant differences in healthy life expectancy among different social classes; groups with higher social classes tend to live longer and healthier lives, and the difference in healthy life expectancy is significantly greater than the difference in life expectancy (9–11).

This study uses the data of the China Health and Retirement Longitudinal Study, takes intrinsic capacity as the health measurement standard, and uses the multistate life table method to measure the healthy life expectancy of the older adult in China to construct health indicators for the older adult that meet international standards and suit China's national conditions and enrich the health connotation of older adult individuals. The aim is to provide a basis for scientific, objective, standardized and operable evaluation of the health level of the older adult in China and to verify and supplement the empirical results of Chinese scholars in the field of healthy life expectancy in older adult individuals. Based on the theory of social stratification, the impact of different social stratification indicators on the health status of older adult individuals is comprehensively analyzed from a dynamic perspective, and the problems of health imbalance in the elder care service system are examined to provide accurate guidance for the government to formulate relevant policies and improve the social system and a reference for the construction of the retirement service system.

## Methods

### Study population

Participants in this study were from the China Health and Retirement Longitudinal Study (CHARLS). The CHARLS collected high-quality longitudinal study data (e.g., demographic, economic, health, retirement) of a nationally representative population of individuals aged 45 and older and their spouses, covering 150 counties and 450 communities (villages) in 28 provinces (autonomous regions and municipalities directly under the central government) (12). The baseline study was conducted in 2011, and follow-up assessments were conducted every 2 years using face-to-face computer-assisted individual interviews. This study uses CHARLS data because it includes details such as basic information, health status and functioning, household information, and financial income. These comprehensive and longitudinal data enable assessment of the intrinsic capacity level of older adult individuals, accurate estimates of healthy life expectancy and analysis of social stratification. In this study, the CHARLS project team applied for authorization to use the data, and it was approved by the Biomedical Ethics Committee of Peking University (approval number: IRB00001052-11015). All participants signed informed consent forms (12).

This study used sociological and health CHARLS data from 2011, 2013 and 2015 (health examination data for the older adult in 2018 is lacking). The CHARLS project is a multistage sample survey, and CHARLS has made weighted structural adjustments to the 2011 baseline respondent information data. The 2013 and 2015 longitudinal tracking survey data weights are based on the baseline weights (or rather, weights with nonresponse adjustments), inverse probability weighting factors are constructed from whether respondents participated in the second wave of logit regression conditional on participation in the baseline, and the longitudinal weights account for the data of deceased respondents. In 2011, there were 17,705 weighted samples, 15,179 weighted samples participated in the 2013 longitudinal follow-up survey, and 14,574 weighted samples participated in the 2015 longitudinal follow-up survey, including 6,053 cases in the population aged 60 and older. The sample (233 people) with missing key variables (gender, education level, place of residence, economic income, etc.) and abnormal intrinsic ability assessment results and the sample (28 people) who did not participate in the intrinsic ability assessment in the 3 surveys were obtained, and the baseline number was 5,792 cases. In the end, the number of people remaining in the 2015 follow-up survey was 4,468.

### Intrinsic capacity assessment

The WHO proposes five dimensions of intrinsic capacity, including motor, cognition, vitality, sensory and psychology, the determination of which is of great significance for objectively measuring the intrinsic capacity of the older adult and comprehensively reflects the health status of the older adult from a holistic perspective. Beard et al. (13) clearly described the components of each domain and examined the structure and predictive validity of the concept of intrinsic capacity. At the same time, the WHO has also developed corresponding assessment tools based on each dimension of intrinsic capacity, providing recommendations and guidelines for its measurement from the perspective of screening and in-depth assessment and elaborating in the publication (ICOPE). In this study, we used the data of the 2011–2015 China Health and Retirement Longitudinal Study. Based on the information available in the database and the tools recommended by WHO ICOPE, this study selected the following methods for the assessment of intrinsic capacity: cognition (temporal orientation, memory and manipulation), motor (gait speed, sit-up test, and balance test), vitality (BMI), sensory (hearing and vision), and psychological (depression).

### Cognition

The cognitive dimension mainly includes the ability to orient time (year, month, day, week, season); memory (listening to ten words, repeating them and recalling them when questioned); and calculation power (100 minus 7). The total score was 30, with ≤ 9 being moderate to severe cognitive impairment, 10–26 being mild to moderate cognitive impairment, and 27–30 being normal (14).

### Motor

The motor dimension mainly includes gait speed, sit-up test and balance test. Each section scored a total of 4 out of 12, with ≤ 2 being classified as moderate to severe impairment of motor function, 3–9 as mild to moderate impairment, and 10–12 as normal (14).

### Vitality

Indicators of vitality function are not clear, and there is no uniform definition. The vitality dimension of this study mainly measured body mass index (BMI). BMI is one of the most commonly used nutritional evaluation indicators in clinical practice, and many foreign studies have used BMI as a single index to investigate the incidence of malnutrition in patients and affirm its clinical value. In this study, BMI was divided into three categories according to Chinese standards, namely thin type (BMI <18.5 kg/m2), normal type (18.5 kg/m2 ≤ BMI <24 kg/m2), and obesity or overweight (BMI ≥ 24 kg/m2).

### Sensory

The sensory dimensions mainly included hearing and visual function, and self-report methods were used to assess hearing and visual function. Vision is mainly measured by asking older adult individuals: “How do you see things in the distance? For example, can you recognize a friend across the street (with or without glasses)?” Hearing is mainly measured by asking older adults, “How is your hearing?” (If you wear hearing aids, how do you hear when you wear hearing aids?) Visual acuity and hearing results are assessed using a 5-point Likert scale, with “1” meaning poor vision and hearing and “5” meaning excellent vision and hearing.

### Psychological

The psychological dimension is a depression assessment that uses a 10-item Chinese version of the depression self-rating scale (CESD). Huang et al. (15) studied the reliability and validity of the scale in the middle-aged and older adult of China, and the results showed that the total Cronbach's α coefficient of the scale was 0.815. The options for each item are composed of 4 levels and assigned as follows: “little or not at all = 0,” “some or little = 1,” “occasional or moderate = 2,” and “most or all = 3.” The total score ranges from 0 to 30 points. Questions 5 and 8 are positive factors, and reverse scoring is needed. A CESD score <10 points is considered to indicate no depressive symptoms, 11–17 points represents possible depressive symptoms, and >18 points indicates depressive symptoms (14, 16).

Given the clinical practical utility of summary scores from routine geriatric assessments, López-Ortiz et al. (14) proposed an intrinsic capacity score accounts for the characteristics that each person possesses as a whole. López-Ortiz et al. (14) argue that composite scores ranging from 0 (worst) to 10 (highest) can be used, which equally weights all five dimensions by establishing a 0–2 range to stratify the state of each of them (i.e., 0 = severely impaired; 1 = partially damaged; 2 = slightly damaged or normal). This study draws on the scoring method of intrinsic capacity in López-Ortiz's study (14), that is, total intrinsic capacity score = motor score + vitality score + cognitive score + psychological score + sensory score, where 1 represented mild impairment and 2 represented no impairment. The total scores of each dimension were added to form a complete intrinsic capacity score, in which 0–4 indicated severe disability or care dependence, 5–8 indicated functional decline, and 9–10 indicated high and stable intrinsic ability (14).

### Multistate life table method

In this study, healthy life expectancy was calculated with the help of IMaCH software. Because IMaCH software is able to fit subjects with missing health status data at only partial time points, it can still fit the probability of healthy transfer measured in the overall sample (17). Therefore, the IMaCH software function was applied in this study, and some samples with missing data at the point in time were not deleted to preserve the integrity of the samples to the greatest extent and ensure that the measurement results were representative (17).

In this study, the multistate life table method was used to measure healthy life expectancy. The multistate life table method is one of the important methods of demography. Healthy life expectancy, which is based on the multistate life table, refers to the average number of years that an individual will live in a healthy state under the current mortality and morbidity estimates. The probability of health transfer is calculated by tracking the transition between different health states, and the average life expectancy is estimated by estimating the length of time in different healthy states (13, 18, 19). This method accounts for the transition between the health status of the study subjects and can more accurately and realistically calculate healthy life expectancy than Sullivan's method (18).

**Figure 1:**
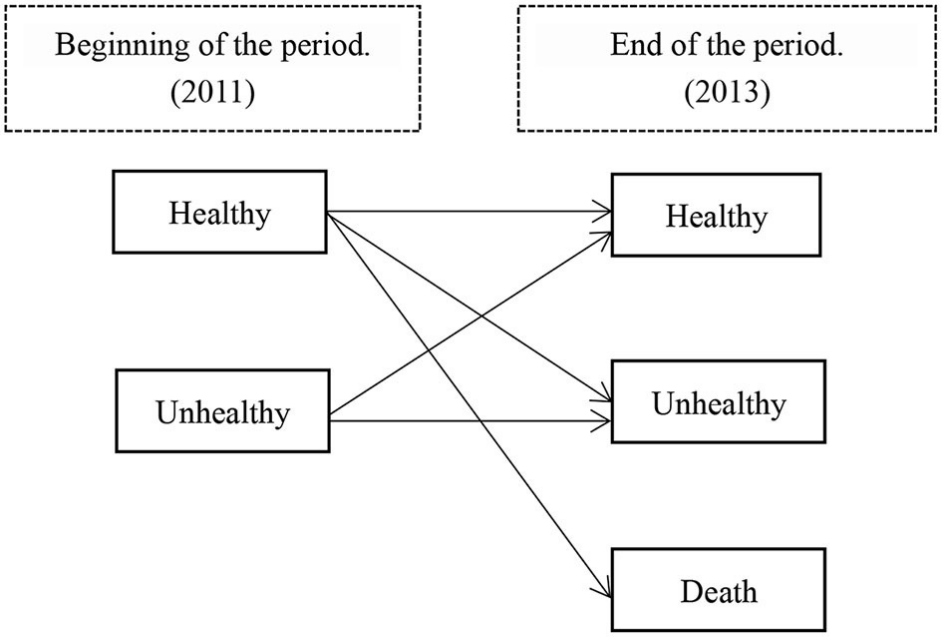
Health status transition diagram.

This study defines health status based on the total intrinsic capacity score. That is, a total score of intrinsic ability ≥9 points is defined as a state of health; a score of 9 < is defined as an unhealthy state. During follow-up, if the participant died, it was defined as a state of death. The death confirmation of the respondent usually requires the representative who has been living with the respondent for a long time to present the respondent's death registration and certificate, which is issued by the hospital or the neighborhood committee where the respondent is registered, which records the respondent's death registration number, time, place, cause of death, and the respondent's previous information. The healthy and unhealthy states are transfer states, and the death state is the absorption state. Figure 1 shows the health transition diagram.

Based on longitudinal data, the operation of the multistate life table method first estimates the probability of health transition according to the change in health status between different time points, and the probability of health state transfer refers to the probability that the individual who is in state ***i*** at moment ***x*** is in state ***j*** at time ***x+n*** later, that is, ${\;}^{i}P_{x,x+n}^{j}$. First, assume that following the Markov chain, the probability that the individual's state at age ***x*** is ***j*** , and the age state of ***x+h*** is ***k*** : $P_{x}^{jk}=P[X(x+h)=k|X(x)=j].$ Second, let $L_{t1-t2}^{\left(i\right)}$ represent the probability ${\;}_{d1}P_{x1}^{jk}$ of the transition of an individual from state ***j*** to state ***k*** between ***t1*** and ***t2*** , and if there are two transformations (from ***j*** to ***k*** and then to ***l*** ), then the probability of state transition of individual ***i*** is $L^{\left(i\right)}={(}_{d1}P_{x1}^{jk})\times {(}_{d2}P_{x2}^{kl})$. The initial state of health status is ***i*** , and the proportion of outcome states 1 (healthy) and 2 (unhealthy), healthy probability and unhealthy probability: Wt  i1(x)=P t x−ti1/P t x−ti1+P t x−ti2;Wt  i2(x)=P t x−ti2/P t x−ti2+P t x−ti2. The incidence of individual outcome status ***j*** was then calculated: ${\;}_{y}P_{x}^{j}(\theta )={\;}_{y}P_{x}^{1j}(\theta )+W^{2}(x,\theta ){[}_{y}P_{x}^{2j}(\theta ){-}_{y}P_{x}^{1j}(\theta )]$.

After obtaining the probability of transition of different states of health, the three states of health, unhealth and death are encoded as ***h*** , ***u*** and ***d***, respectively, and a first-order matrix of the probability of transition between different states of health and the matrix of the number of surviving people are constructed (20).


$$\begin{array}{l} P_{x,x+t}={(}^{i}P_{x,x+n}^{j})=\left[\begin{array}{ccc} {\;}^{h}P^{h}{\;}_{x,x+n} & {\;}^{h}P^{u}{\;}_{x,x+n} & {\;}^{h}P^{d}{\;}_{x,x+n}\\ {\;}^{u}P^{h}{\;}_{x,x+n} & {\;}^{u}P^{u}{\;}_{x,x+n} & {\;}^{u}P^{d}{\;}_{x,x+n}\\ 0 & 0 & 1 \end{array}\right]\\ \;l_{x,x+t}=\left[\begin{array}{ccc} {\;}^{h}l^{h}{\;}_{x,x+n} & 0 & 0\\ 0 & {\;}^{h}l^{h}{\;}_{x,x+n} & 0\\ 0 & 0 & 0 \end{array}\right] \end{array}$$

Based on the transfer probability matrix and the surviving number matrix, the multistate life table calculation formula is used to calculate the healthy life expectancy. ***X*** is age, ***h*** is healthy, ***u*** is unhealthy, ***d*** is death, and ***n*** is follow-up. This study was conducted over a 4-year period.

***l***_***x***_ is the total number of people aged *X*, $l_{x}^{h}$ is the healthy number of years X, $l_{x}^{u}$ is the unhealthy number of years *X*, the number of years ***x-n*** is ***l***_***x−n***_, the healthy number of years ***x-n*** is $l_{x-n}^{h}$, and the unhealthy number of years ***x-n*** is $l_{x-n}^{u}$. ***lx*** is the number of years of survival, $L_{x}^{h}$ is the number of years of healthy survival, and $L_{x}^{u}$ is the number of years of unhealthy survival. ***Tx*** is the cumulative number of years of survival, $T_{x}^{h}$ is the cumulative number of years of healthy survival, and $T_{x}^{u}$ is the cumulative number of years of unhealthy survival. P h x−nh represents the probability that a person who is healthy at age ***x-n*** will remain in a healthy state at age ***x*** ; P h x−nu represents the probability that a person whose state is healthy at age ***x-n*** will change to an unhealthy state at age ***x*** ; P h x−nd represents the probability that a person whose state is healthy at age ***x-n*** will change to death at age ***x*** ; P u x−nh represents the probability that a person whose state is unhealthy at age ***x-n*** will change to a healthy state at age ***x*** ; P u x−nu represents the probability that a person whose state is unhealthy at age ***x-n*** will change to an unhealthy state at age ***x*** ; P u x−nd represents the probability that a person whose state is unhealthy at age ***x-n*** will change to death by the age of ***x*** .


                             lx=lxh+lxulxh=lx-nh-(lx-nh×P h x-nu)-(lx-nh×P h x-nd)                       +(lx-nu×uPx-nh)lxu=lx-nu-(lx-nu×P u x-nh)-(lx-nu×P u x-nd)                      +(lx-nh×hPx-nu)                      Lx=(lx+lx-n)                      Lxh=(lxh+lx-nh)                      Lxu=(lxu+lx-nu)     Tx=∑x=60MaxLx(x=60,64,68,72…,100            +for the highest age group)    Txh=∑x=60MaxLxh(x=60,64,68,72…,100           +for the highest age group)Txu=∑x=60MaxLxu(x=60,64,68,72…,100         +for the highest age group)

$$\begin{array}{l} \begin{array}{c} \text{Life expectancy: }{\text{e}}_{\text{x }=Tx/lx}\\ \text{Healthy life expectancy: }{\text{HE}}_{\text{x}}=T_{x}^{h}/l_{x}^{h} \end{array} \end{array}$$

### Social stratification analysis

Based on Weber's theory of social stratification and the experience of previous researchers, this study explores the differences in healthy life expectancy among older adult individuals with five different social characteristics: age, gender, place of residence, income, and education level, and it makes a comprehensive judgment on the social status factors of health inequality. Study subjects were asked, “Where do you mainly live?,” “What is your average annual income?,” and “What is your level of education?” In this study, considering the visibility of the analysis results and the research patterns in the literature, the responses to some questions were integrated. During follow-up analyses, missing values were considered lost. Table 1 describes the variable settings.

**Table 1:** Variable settings.

**Items**	**Variables**	**Variables explained in the questionnaire**
Demography variable	Gender	“male” = 1, “female” = 2
	Age (year old)	“60–63” = 1, “64–67” = 2, “68–71” = 3, “72–75” = 4, “76–79” = 5, “≥80” = 6
Social stratification	Income	“Low income” = 1, “middle-high income” = 2
	Place of residence	“Town (town, urban and rural or combined towns and townships)” = 1, “rural” = 2
	Education level	“Primary school level and less” = 1, “Middle school level and more” = 2

### Statistical methods

SPSS 25.0 software was used for analysis, the measurement data were expressed as the mean ± standard deviation (*x* ± *s*), and the chi-square test and rank sum test were used for the between-group comparison of baseline data. The *t* test was used for normally distributed data, and the nonparametric test was used for nonnormally distributed data. *P* < 0.05 was considered statistically significant. The preparation of life tables and the calculation of healthy life expectancy under different social stratifications was recorded in Excel and facilitated the comprehensive comparison of the healthy life expectancy of older adult individuals with different social statuses. Finally, an explanatory conclusion was drawn.

## Results

### Basic information

A total of 5,792 older adult aged 60 years and older who participated in intrinsic capacity assessment were included as baseline survey participants, and a total of 4,468 people participated in tracking and surviving in 2015. Among them, 92.01% of older adult were under 80 years old in 2011 and 94.11% in 2015. With the occurrence of deaths, the proportion of older adult in this cohort gradually decreased. In the two tracking datasets, the proportion of males was higher than that of females, which was consistent with the law of the sex ratio of the older adult (7). The research subjects were mainly older adult rural residents, the education level of older adult was concentrated at the level of primary school and less, and most of the interviewed older adult had low incomes. In terms of health status, the proportion of healthy older adult decreased during the follow-up period. Basic information and health transfer status are shown in Table 2.

**Table 2:** Basic information and health transfer status of the study subjects.

**Items**	**2011 baseline survey frequency (%)**	**2013 follow-up** **survey frequency (%)**	**2015 follow-up survey frequency (%)**
**Age group (year)**			
60~	1,403 (24.22)	1,318 (25.83)	1,226 (27.44)
64~	1,260 (21.75)	1,152 (22.57)	1,029 (23.03)
68~	1,073 (18.53)	957 (18.75)	831 (18.60)
72~	827 (14.28)	713 (13.98)	615 (13.76)
76~	766 (13.23)	638 (12.50)	504 (11.28)
80~	463 (7.99)	325 (6.37)	263 (5.89)
**Gender**			
Male	3,334 (57.56)	2,981 (58.42)	2,663 (59.60)
Female	2,458 (42.44)	2,122 (41.58)	1,805 (40.40)
**Place of residence**			
City and town	2,637 (45.53)	2,408 (47.19)	2,117 (47.38)
Rural	3,155 (54.47)	2,695 (52.81)	2,351 (52.62)
**Level of education**			
Primary school and less	3,016 (52.07)	2,743 (53.75)	2,254 (50.45)
Middle school and more	2,776 (47.93)	2,360 (46.25)	2,214 (49.55)
**Income**			
Low income	3,519 (60.76)	3,048 (59.73)	2,659 (59.51)
Middle-high income	2,273 (39.24)	2,055 (40.27)	1,809 (40.49)
**Health status**			
Healthy	1,914 (33.05)	1,656 (33.45)	1,437 (32.16)
Unhealthy	3,878 (66.95)	3,447 (67.55)	3,031 (67.84)
Died	0	407 (7.03)	413 (7.13)
Lost to follow-up	0	282 (4.87)	222 (3.83)

### Healthy life expectancy of older persons in different social stratifications

In this study, the differences in healthy life expectancy among older adult individuals in different social strata were explored from five aspects: age, gender, place of residence, economic income, and education level. The results are mainly presented as life expectancy (LE), healthy life expectancy (HLE), and the proportion of healthy life expectancy to average life expectancy.

Table 3 shows the average life expectancy and healthy life expectancy of the older adult by age and sex in China. The calculation results show that the average life expectancy of older adult in China at age 60 is 21.07 more years, the healthy life expectancy is 16.89 more years, and the healthy life expectancy accounts for 80.2%. With increasing age, the health status of the older adult in China is gradually declining in the process of aging. In terms of gender, the average life expectancy of men at age 60 is 20.36 more years, the healthy life expectancy is 17.16 more years, and the proportion of healthy life expectancy is 84.3%. The average life expectancy of women at age 60 is 20.70 more years, the healthy life expectancy is 16.64 more years, and the proportion of healthy life expectancy is 80.4%. The data show that women's average life expectancy is higher than that of men, but their healthy life expectancy is significantly lower than that of men, indicating that older women, although living longer, do not have as many healthy years as men.

**Table 3:** Average life expectancy and healthy life expectancy of the older adult by age and sex in China (years).

	**Overall**			**Male**			**Female**		
**Age group (year)**	**LE**	**HLE**	**HLE/LE (%)**	**LE**	**HLE**	**HLE/LE (%)**	**LE**	**HLE**	**HLE/LE (%)**
60~	21.07	16.89	80.2	20.36	17.16	84.3	20.70	16.64	80.4
64~	18.73	13.14	70.2	16.76	13.55	80.8	16.89	12.88	76.3
68~	14.98	10.02	66.9	12.60	9.58	76.0	13.65	8.98	65.8
72~	9.32	6.11	65.6	9.98	6.50	67.1	11.73	5.67	48.3
76~	6.29	3.88	61.7	5.56	3.44	61.9	6.88	3.00	43.6
80~	2.23	1.11	49.8	3.08	1.54	50.0	3.72	1.30	34.9

As shown in Table 4, gender, income and education level were used as control variables to measure the average life expectancy, healthy life expectancy and proportion of older adult individuals with different education levels. At age 60, the average life expectancy of older adult rural residents in China is 19.53 more years, the healthy life expectancy is 15.30 more years, and the proportion of healthy life expectancy is 78.3%, while the average life expectancy of older adult urban residents in China at age 60 is 20.84 more years, the healthy life expectancy is 16.17 more years, and the healthy life expectancy accounts for 77.6%. As shown in Table 4, these data show the rural–urban difference in healthy life expectancy among older adult individuals. The average life expectancy and healthy life expectancy of urban older adult are higher than those of rural older adult individuals, but the proportion of healthy life expectancy is lower than that of rural older adult.

**Table 4:** Average life expectancy and healthy life expectancy of the older adult in China by urban and rural areas.

	**Rural**			**Urban**		
**Age group (year)**	**LE**	**HLE**	**HLE/LE (%)**	**LE**	**HLE**	**HLE/LE (%)**
60~	19.53	15.30	78.3	20.84	16.17	77.6
64~	16.78	12.46	74.3	17.24	12.68	73.5
68~	11.64	8.24	70.8	13.42	8.90	66.3
72~	9.49	6.03	63.5	9.79	6.16	62.9
76~	6.98	4.08	58.5	7.48	4.23	56.6
80~	3.93	1.50	38.2	4.67	1.67	35.8

As shown in Table 5, gender, urban and rural areas, and education level were used as control variables to measure average life expectancy, healthy life expectancy and proportion of older adult at different education levels. At age 60, the average life expectancy of low-income older adult in China is 20.22 more years, the healthy life expectancy is 15.19 more years, and the healthy life expectancy accounts for 75.1%, while the average life expectancy of middle- and high-income older adult in China is 20.76 more years, the healthy life expectancy is 16.26 more years, and the healthy life expectancy accounts for 78.3%. These data show the impact of income on the healthy life expectancy of older adult. The proportions of average life expectancy, healthy life expectancy and healthy life expectancy of middle- and high-income older adult are higher than those of low-income older adult, indicating that the health status and quality of life of high-income older adult are better than those of low-income older adult.

**Table 5:** Average life expectancy and healthy life expectancy of older adult individuals by income level in China (years).

	**Low-income**			**Middle-high income**		
**Age group (year)**	**LE**	**HLE**	**HLE/LE (%)**	**LE**	**HLE**	**HLE/LE (%)**
60~	20.22	15.19	75.1	20.76	16.26	78.3
64~	17.35	12.73	73.4	18.64	13.83	74.2
68~	12.66	8.32	65.7	14.52	9.89	68.1
72~	8.36	4.65	55.6	10.88	6.20	57.0
76~	6.92	3.43	49.6	7.01	3.67	52.3
80~	3.07	1.25	40.7	5.34	2.50	46.8

As shown in Table 6, gender, urban and rural residence, and income were used as control variables to calculate the average life expectancy, healthy life expectancy and proportion of the older adult at different levels of education. At age 60, the average life expectancy of the older adult with a primary school education level and below in China is 21.74 more years, the healthy life expectancy is 16.42 more years, and the proportion of healthy life expectancy is 75.5%, while the average life expectancy of the older adult with a middle school education level over age 60 is 22.84 more years, the healthy life expectancy is 17.15 more years, and the healthy life expectancy accounts for 75.1%. These data show that the average and healthy life expectancy of older people with lower levels of education is longer than that of older people with higher levels of education, but the proportion of healthy life expectancy is relatively high.

**Table 6:** Average life expectancy and healthy life expectancy of the older adult by education level in China (years).

	**Primary school and less**			**Middle school and more**		
**Age group (years)**	**LE**	**HLE**	**HLE/LE (%)**	**LE**	**HLE**	**HLE/LE (%)**
60~	21.74	16.42	75.5	22.84	17.15	75.1
64~	18.87	13.83	73.3	20.53	14.54	70.8
68~	14.60	10.12	69.3	16.21	11.20	69.1
72~	11.89	8.13	68.4	12.41	8.40	67.7
76~	8.97	4.29	47.8	9.84	4.43	45.0
80~	5.95	2.00	33.6	7.21	2.30	31.9

## Discussion

With the development of the social economy, people's demand for health is increasing, and the understanding of health is more in depth. The selection of healthy life expectancy indicators not only includes objective measurement indicators such as disability and ADLs but also includes subjective measurement indicators based on self-assessment of health, which can reflect the development level of healthy life expectancy of the general population in China. However, with the acceleration of the aging process, the health of the older adult has received increasing attention, and the Global Report on Aging and Health released by the WHO in 2015 (5) promoted healthy aging as a strategic measure to cope with population aging worldwide. Intrinsic capacity is the core of healthy aging, enriching the connotation of the health of older adult.

This study is the first to use intrinsic capacity as a health standard to measure the average life expectancy and healthy life expectancy of the older adult in China. According to data released by the Department of Aging Health of the National Health Commission (21), the life expectancy of 60-year-old people in China is 21.04 years, while the healthy life expectancy is 16.42 years. The life expectancy of 60-year-old people in China measured by this study is 21.07 years, and the healthy life expectancy is 16.89 years, which is close to the data released by the National Health Commission, indicating that the intrinsic capacity as a health evaluation index to measure the healthy life expectancy of the older adult has certain reference value. As a multidimensional and relatively strict evaluation index, the intrinsic capacity fully reflects the reserve function of the older adult and is committed to improving the quality of life and extending the healthy life expectancy of older adult.

In the past, China usually adopted a disease-centered nursing model for older adult, believing that the older adult are healthy without disease, thus proposing the concept of disease-free healthy life expectancy. Liu et al. (22) used the 2011 CHARLS data to measure the disease-free healthy life expectancy of older adult in China, and the results showed that the disease-free healthy life expectancy of the 60-year-old group was only 3.2 more years. Disease-free healthy life expectancy is expressed as life expectancy in a healthy state without certain disease, and as the older adult age, the proportion who suffer from chronic diseases increases; the 2022 14th 5-Year Plan for Healthy Aging shows that more than 78% of the older adult in China have at least one chronic disease (23). At present, most chronic diseases can be prevented and controlled by lifestyle changes. Kong et al. (24) found that lifestyle care management can effectively improve medical compliance behavior, reduce the occurrence of chronic disease complications, and thus improve the quality of life of patients. Therefore, using disease as a determinant of health will increase the number of unhealthy people, resulting in a longer unhealthy life expectancy and a shorter healthy life expectancy. Thus, discussing health in terms of disease no longer meets the health needs of older adult.

In 2001, the WHO published the International Classification of Functioning, Disability and Health, and scholars from various countries began to use disability or function as a health indicator to measure the healthy life expectancy of older adult. Wang et al. (25) estimated the healthy life expectancy of older adult aged 60 and older in Jiangxi Province, China, in 2018, and the results showed that the disability-free healthy life expectancy of older adult aged 60 was 18.12 more years, which was significantly higher than the results of this study. Disability-free healthy life expectancy evaluates the health status of a population by combining the disability status of a population with life expectancy. In 2006, China conducted a large-scale national sample survey of persons with disabilities, which not only classified disabilities into visual, hearing, speech, physical, intellectual, mental, and multiple disabilities but also graded each type of disability according to the degree of impairment or abnormality (26, 27). Because the determination of disability and disability level is an objective survey based on medical testing standards and even the use of professional equipment by doctors (27), China's disability statistics are relatively low; according to the survey, the total disability prevalence rate of the population ages 60 and older is 24% (28). Although older adult have defective body parts, if these defects do not affect physical functioning, it is not comprehensive to judge their health based on whether they have physical disabilities.

At present, the most commonly used health indicator in China is the ability to perform ADLs. This indicator was originally proposed by Katz, who paid more attention to the physical ability of older adult, and then Lowton et al. (29) extended the concept of ADLs by introducing the ability to perform instrumental activities of daily living (IADLs); both of these indicators are now used to evaluate the ability of older adult to live independently. Wen et al. (30), based on the 2013–2018 CHARLS data, used ADL as health standards to measure the healthy life expectancy of older adult, and the results indicated that under the ADL standard, the healthy life expectancy of the 60-year-old group in China was 19.8 years. The data show that the healthy life expectancy measured by ADL standards is higher than the results of this study, and the reasons for the differences between different research results are analyzed. First, because ADL mainly measures the physical function of older adult individuals, compared with the assessment of intrinsic capacity, its standard of assessing health is relatively simple, so the number of healthy people with ADL as the standard is significantly more than the healthy number of healthy people with intrinsic capacity as the standard. At the same time, ADL limitations are usually observed when functional decline is obvious, and the decline in intrinsic capacity, a sensitive dynamic indicator of healthy aging, tends to occur before or even earlier than the onset of age-related diseases or symptoms.

Although the reliability and validity of objective measurement indicators such as disease, disability, ADL, etc., are high, it is difficult to measure large-scale populations, so people expect to find a subjective measurement and a method that can be close to objective health measurement, and some studies believe that health self-assessment is a comprehensive evaluation index that can evaluate all health states (31). Based on the CHARLS data from 2011 to 2015, Huang et al. (17) calculated the healthy life expectancy of older adult in China with self-rated health as the standard, and the results showed that the healthy life expectancy of the 60-year-old group of older adult in China was 16.2 years. This is lower than the findings of this study, possibly because people self-evaluate their health status by considering lifestyle, disease burden, mental state, social, physical, and emotional factors, and specific cultural factors. Because Chinese people are deeply influenced by the golden mean, they tend to be more neutral in self-rated health options and are more subtle in expressing their opinions than people in Western countries. Second, self-assessment of health largely depends on the subjective empirical perception of the disease in older adult, and many older adult may subjectively rate their health lower than physical activity status (31).

The results of this study found that the life expectancy of older men is lower than that of women, but the healthy life expectancy of older men is significantly higher than that of women, consistent with most findings. Oyen et al. (32) proposed the male and female sexual health-survival paradox, that is, women have worse health than men, but women are less likely to die than men of the same age, thus showing that women have a longer life expectancy than men, but their healthy life expectancy is lower than that of men. Song et al. (33), based on the analysis of healthy life expectancy and its proportion of total life expectancy, concluded that the value for older adult men is always higher than that of women, and being female and older adult poses a significant health disadvantage. Gender differences in life expectancy include physiological factors and behavioral patterns. Physiologically, it is believed that women have positive immune function, the protective effect of estrogen, and the compensatory effect of the second X chromosome, which can reduce the activity of growth hormone and the insulin-like growth factor 1 signaling cascade, as well as the effect of oxidative stress on aging and disease (34). Second, in terms of behavior, women have lower rates of smoking and drinking and more social activities. Although the incidence of physical illness is higher than that of men, women are more likely to seek medical attention on their own initiative and use health services more frequently. Many of these factors contribute to the lower risk of death in women than in men. However, the study found that women's healthy life expectancy is less than that of men because women are more likely than men to suffer from nonfatal chronic diseases, which, while contributing less to the risk of death, contribute to women's poor health and longer survival from the disease. At the same time, due to the different social division of labor, most women still have to bear heavy housework and family care after retirement, and their health is far worse than that of older men.

The results of this study found that the life expectancy and healthy life expectancy of older adult living in urban areas are significantly higher than those of older adult living in rural areas, but the proportion of healthy life expectancy is lower than that of older adult living in rural areas, which is inconsistent with some of the research results. Liu et al. (35) discussed the trend of self-measured healthy life expectancy and analyzed the differences between urban and rural areas and found that the life expectancy and self-measured healthy life expectancy of urban residents of all ages and their proportions were higher than those of rural residents. Huang et al. (17) measured the healthy life expectancy of the older adult in China by self-rated health as an index and found that the health status of the urban older adult was significantly better than that of the rural older adult; not only was the average life expectancy longer than that of the rural older adult, but the healthy life expectancy was also longer, and the proportion of healthy life expectancy was also higher. China has long had a dual urban–rural structure, the quality of rural health services is lower than that of cities, and the medical environment facilities are relatively underdeveloped, making it difficult to provide relatively sound health and medical services for older adult in rural areas. Second, the education level of the rural older adult is lower than that of the urban older adult; the level of health education is low, and the health awareness is weak, which causes the life expectancy and healthy life expectancy of the urban older adult to be higher than that of the rural older adult. However, the results of this study also found that the proportion of healthy life expectancy of urban older adult is lower than that of rural older adult, and the reason for this phenomenon may be that the economic income of rural older adult mainly comes from the primary industry, high work intensity, and mainly physical exertion, which potentially leads to the duration of the health state of rural older adult individuals being longer than that of urban older adult. The healthy life expectancy of rural older adult under the original health conditions is longer than that of urban older adult, and rural older adult live shorter lives than urban older adult in an unhealthy state. Conversely, in urban areas, good medical conditions and living conditions protect unhealthy older people, resulting in a relatively high risk of death and extending their overall life expectancy when they are unhealthy.

The results of this study found that the life expectancy, healthy life expectancy and proportion of middle- and upper-income older adult are significantly higher than those of lower-income older people, which is consistent with most research results. Kaneda et al. (36) showed that the income of the male group had a significant impact on their healthy life expectancy, and the healthy life expectancy of the high-income group was 1.57 times that of the low-income group, but there was no significant difference in healthy life expectancy between the high-income group and the low-income group of women. Qiao et al. (37) compared and analyzed the population health indicators among 31 provinces in China and found that the health level of older adult individuals with good socioeconomic conditions was significantly higher than that of older adult with poor economic conditions. Older adult with low incomes cannot flexibly access a variety of health resources through multiple strategies and channels, are limited by economic conditions and are prone to the phenomenon of “minor diseases not treated,” and the pursuit of healthy quality of life is low.

The results of this study found that the life expectancy and healthy life expectancy of older adult with higher education were significantly higher than those of older adult with low education, but the proportion of healthy life expectancy was relatively low, which is consistent with some research results. Jiao et al. (38) found that older people with lower levels of education have lower life expectancy and healthy life expectancy than older people with higher levels of education. However, in terms of healthy life expectancy as a proportion of total life expectancy, older people with lower levels of education are higher than those with higher levels of education. Wu et al. (7) used ADL as an indicator to measure the healthy life expectancy of the older adult in China and found that the average life expectancy and healthy life expectancy of the older adult with higher education levels were longer, but the proportion of healthy life expectancy was relatively low. The findings are the same as the difference in healthy life expectancy between urban and rural older people. Older adult with low education levels are mostly physical laborers, and their physical function is relatively good, so in the initial healthy state, their health is expected to be maintained for a relatively long time, but due to the limitations of economic level, older adult individuals with low education levels have a lower functional recovery rate and a higher risk of death, resulting in relatively short unhealthy survival. Conversely, older adults with higher levels of education have higher rates of functional recovery and lower risk of death, and this group is expected to transition to unhealthy for a longer period of time than less educated older people under initially healthy conditions and remain unhealthy under unhealthy conditions for a higher expected period of time than less educated older people, ultimately resulting in a relatively longer overall unhealthy life expectancy for older people with higher levels of education.

The root cause of the health inequality of China's older adult is unequal access to health resources for different social classes. Factors such as living conditions and health services determined by social class are variables related to health level, and these variables should be observed not only in old age but also in the entire life course of the individual (39). In the current social equity-oriented social context and in the global context of promoting healthy aging, we must take active and effective measures to reduce health inequalities caused by social class differences.

Although the current policy benefits for the older adult in society are gender neutral, they ignore the different social roles, responsibilities and abilities of men and women and subtly exacerbate gender health inequalities. Therefore, the government should account for gender differences when formulating public policies and allocating public resources. The focus on promoting women's rights and equality throughout the life cycle requires improving women's disadvantage in nutrition, education, and health services early on and reducing disparities in employment, income, and family care in adulthood to enhance women's health and socioeconomic accumulation throughout their lives (40, 41).

The basic health rights and interests of older adult from low social strata should be guaranteed, including rural older adult with low education levels and poor economic conditions at the bottom of China's basic social security system. At this stage, there are still inequalities in the level of medical services, pension policies and educational resources in China, and the government should actively carry out the reform of the medical and health system to form a fair and healthy medical mechanism for the whole society (42). The government should actively narrow the gap between urban and rural areas, promote the construction of the older adult health service system and older adult health services as an important component of the construction of local health service systems and health development plans, and promote the balanced development of urban and rural and regional older adult health services. At the same time, it is also necessary to actively improve the aging level of the medical and health service system, improve the older adult health service system, promote the older adult health prevention threshold forward, continue to expand the coverage of high-quality community older adult health services in terms of content, region, infrastructure and other aspects, and provide precision health services to the older adult with different intrinsic capacities (43).

The awareness of health services for the older adult should be enhanced, publicity and education on health knowledge for the older adult should be strengthened, and a variety of methods and media should be used to widely disseminate popular science knowledge such as nutritious diet, sports and fitness, mental health, disease prevention, rational drug use, rehabilitation nursing, life education and health care to the older adult and their caregivers. Health literacy promotion projects for the older adult should be organized and implemented to strengthen health education in a targeted manner and improve the health literacy of older adult. Regular health check-ups should be conducted and the health status of the older adult in the lower social class considered in the provision of high-quality medical protection.

The advantage of this study is that it adopts prospective cohort studies and, for the first time, uses intrinsic capacity as an indicator to measure the health of the older adult by measuring healthy life expectancy, using intrinsic capacity as a framework for assessing and monitoring the health of older adult. Different from the previous concept of discussing health by the presence of diseases, this perspective pays more attention to the function of older adult and measures the healthy life expectancy of the older adult with intrinsic capacity. This forms a new health standard by which to measure healthy life expectancy and expands the theoretical framework of health evaluation connotation. Furthermore, we used the multistate life table method to measure and analyze the difference in the healthy life expectancy of the older adult at different social levels, which not only reflects the changes in the health status of the older adult but also provides a reference for the formulation of national policies. The study was also flawed. First, Because the physical examination data were not published in the CHARLS database in 2018, this study could not use the latest updated follow-up data in 2018 to study the healthy life expectancy of older adult individuals, resulting in the untimeliness of this study. The team will continue to pay attention to the latest news on CHARLS data in 2023 and will continue to conduct further research on the healthy life expectancy of older adult individuals by expanding the sample size and extending the follow-up time; furthermore, the latest data will be used to discuss and analyze the current status of the intrinsic capacity of older adult and the empirical results of the healthy life expectancy of older adult in communities in China based on the intrinsic capacity health standard. Second, intrinsic capacity reflects the overall function of the body and mind, and a reasonable scoring method can better reflect the functional level of older adult and enhance the comparability of different research results. The scoring method of intrinsic capacity is still in the exploratory stage. In this study, only the integrity of intrinsic capacity was used as a health criterion for the older adult, and the decline in intrinsic capacity was considered an unhealthy criterion. Therefore, how to translate each individual function into the scoring method for overall intrinsic capability needs further exploration. Third, older persons are a special group, and existing social stratification standards have certain limitations for them. For example, most people aged 60 and older have withdrawn from the labor market or have become inactive. Therefore, whether the economic income level of the older adult can become the criterion for social stratification needs further confirmation.

## Conclusion

Healthy life expectancy combines life length and quality of life and is a comprehensive index used to evaluate the health level of the world population. The health level of the older adult is the basic factor in the construction of a social old-age service system, and the health inequality caused by social stratification is an important perspective when discussing healthy life expectancy. To this end, in today's sharing of the fruits of social and economic development, the government and all levels of society should focus on the health status of older adult in the lower social class, provide them with more health knowledge and medical services through different channels, and protect their health rights and interests. As a framework for monitoring the health of older adult, intrinsic capacity scientifically, objectively and comprehensively evaluates the health level of the older adult in various dimensions and enriches the connotation of health among these individuals in China. Future research can synthesize advanced research concepts at home and abroad and combine China's national conditions to propose a more scientific intrinsic capacity evaluation system and objectively assign weights in various fields to more accurately measure the healthy life expectancy of older adult.

## Data availability statement

The datasets presented in this study can be found in online repositories. The names of the repository/repositories and accession number(s) can be found in the article/supplementary material.

## Ethics statement

The studies involving humans were approved by the public database of the China Health and Retirement Longitudinal Study and received ethics approval from the Biomedical Ethics Committee of Peking University. The studies were conducted in accordance with the local legislation and institutional requirements. The participants provided their written informed consent to participate in this study.

## Author contributions

ML: Conceptualization, Data curation, Investigation, Writing – original draft, Writing – review & editing. MZ: Writing – review & editing. LZ: Funding acquisition, Resources, Supervision, Writing – original draft, Writing – review & editing. JZ: Writing – review & editing. NS: Writing – review & editing.
